# Supplementary material for: Circulating Cell-Free DNA and RNA Analysis as Liquid Biopsy: Optimal Centrifugation Protocol
Source: Cancers (Basel). 2019 Mar 30;11(4):458. doi: 10.3390/cancers11040458 (PMC6521186; doi:10.3390/cancers11040458)
Supplement: Supplementary file 1 [file cancers-11-00458-s001.pdf]

Supplementary Materials: Circulating Cell-Free DNA and RNA Analysis as Liquid Biopsy: Optimal Centrifugation Protocol

Laure Sorber, Karen Zwaenepoel, Julie Jacobs, Koen De Winne, Sofie Goethals, Pablo Reclusa, Kaat Van Casteren, Elien Augustus, Filip Lardon, Geert Roeyen, Marc Peeters, Jan Van Meerbeeck, Christian Rolfo and Patrick Pauwels

Table S1. Statistical analysis of total cfDNA.

**cfDNA**	**EDTA**						**Streck**					
	**CP**	**AdBasic_P**	**AdBasic**	**Plat**	**CEN**	**AdCEN**	**CP**	**AdBasic_P**	**AdBasic**	**Plat**	**Streck**	**AdCEN**
**Copies/mL**	**Basic**	0.956	**0.001**	**0.000123**	**0.000311**	**0.000222**	**Basic**	0.168	**0.001**	**0.001**	**0.002**	**0.002**
	**AdBasic_P**		**0.000278**	**0.00006**	**0.000139**	**0.000097**	**AdBasic_P**		0.383	0.291	0.491	0.358
	**AdBasic**			0.715	0.651	0.930	**AdBasic**			0.312	0.679	0.818
	**Plat**				0.599	0.559	**Plat**				0.550	0.635
	**CEN**					0.965	**Streck**					0.836
**Cp *LINE-1***	**Basic**	0.474	**0.004**	**0.002**	**0.003**	**0.003**	**Basic**	**0.012**	**0.043**	**0.013**	0.28	0.27
	**AdBasic_P**		**0.016**	**0.016**	**0.019**	**0.026**	**AdBasic_P**		0.108	0.098	**0.043**	**0.031**
	**AdBasic**			0.849	0.827	0.965	**AdBasic**			0.435	0.251	0.312
	**Plat**				0.77	0.715	**Plat**				0.085	0.081
	**CEN**					0.977	**Streck**					0.963
**% long fragments**	**Basic**	**0.000164**	**0.000002**	**1.36E−07**	**1.36E−07**	**1.36E−07**	**Basic**	**0.000127**	**0.024**	**0.004**	**0.001**	**0.000338**
	**AdBasic_P**		**1.13E−07**	**9.59E−08**	**9.59E−08**	**9.59E−08**	**AdBasic_P**		**0.000008**	**0.000006**	**0.000006**	**0.000006**
	**AdBasic**			**0.003**	**0.00011**	**0.00011**	**AdBasic**			0.358	**0.035**	**0.031**
	**Plat**				0.358	0.35	**Plat**				0.215	0.154
	**CEN**					0.827	**Streck**					0.748

CP: centrifugation protocol; numbers in bold: statistically significant ($p < 0.05$).

Table S2. cfDNA integrity of EDTA vs Streck tubes.

CP	Basic	AdBasic_P	AdBasic	Plat	AdCEN
**EDTA vs Streck**	**0.001**	0.085	**0.002**	**0.003**	**0.011**

CP: centrifugation protocol; numbers in bold: statistically significant ($p < 0.05$).

Table S3. Statistical analysis of total cfRNA in EDTA tubes.

	CP	AdBasic_P	AdBasic	Plat	Plat_P	CEN	AdCEN
**cfRNA (copies/mL)**	**Basic**	**0.001**	**0.005**	**0.000387**	**0.006**	**0.000012**	**0.000012**
	**AdBasic_P**		0.333	0.972	**0.000001**	**0.012**	**0.014**
	**AdBasic**			0.361	**0.000002**	**0.006**	**0.004**
	**Plat**				**0.000001**	**0.000437**	**0.001**
	**Plat_P**					**6.38E−07**	**6.39E−07**
	**CEN**						0.843

CP: centrifugation protocol; numbers in bold: statistically significant ($p < 0.05$).
